# Nanomaterials and their composite scaffolds for photothermal therapy and tissue engineering applications

**DOI:** 10.1080/14686996.2021.1924044

**Published:** 2021-06-04

**Authors:** Rui Sun, Huajian Chen, Linawati Sutrisno, Naoki Kawazoe, Guoping Chen

**Affiliations:** a Research Center for Functional Materials, National Institute for Materials Science, Tsukuba, Ibaraki, Japan; b Department of Materials Science and Engineering, Graduate School of Pure and Applied Sciences, University of Tsukuba, Tsukuba, Ibaraki, Japan

**Keywords:** Nanoparticles, magnetic nanoparticles, gold nanoparticles, black phosphorus nanosheets, composite scaffolds, photothermal therapy, tissue engineering, 30 Bio-inspired and biomedical materials, 211 Scaffold / Tissue engineering/Drug delivery, 102 Porous / Nanoporous / Nanostructured materials, photothermal therapy, tissue regeneration

## Abstract

Photothermal therapy (PTT) has attracted broad attention as a promising method for cancer therapy with less severe side effects than conventional radiation therapy, chemotherapy and surgical resection. PTT relies on the photoconversion capacity of photothermal agents (PTAs), and a wide variety of nanomaterials have been employed as PTAs for cancer therapy due to their excellent photothermal properties. The PTAs are systematically or locally administered and become enriched in cancer cells to increase ablation efficiency. In recent years, PTAs and three-dimensional scaffolds have been hybridized to realize the local delivery of PTAs for the repeated ablation of cancer cells. Meanwhile, the composite scaffolds can stimulate the reconstruction and regeneration of the functional tissues and organs after ablation of cancer cells. A variety of composite scaffolds of photothermal nanomaterials have been prepared to combine the advantages of different modalities to maximize their therapeutic efficacy with minimal side effects. The synergistic effects make the composite scaffolds attractive for biomedical applications. This review summarizes these latest advances and discusses the future prospects.

## Graphical abstract

**Figure:**
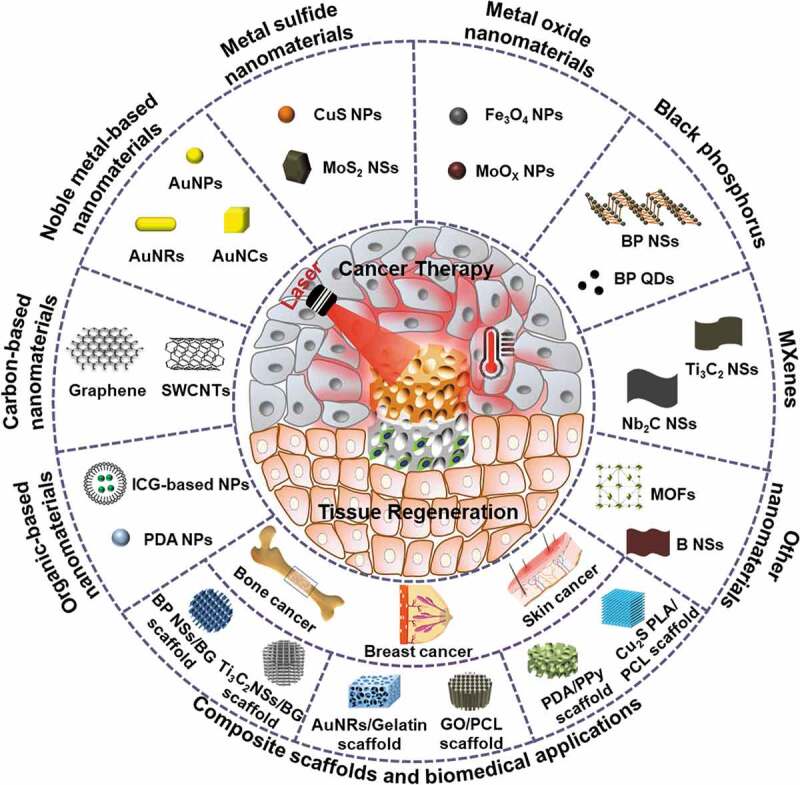

## 1. Introduction

Currently, cancer is a major health problem due to its high morbidity and mortality [1]. Traditional treatments of cancers include surgical resection, chemotherapy, and radiation therapy [2]. However, these treatments have some associated problems. Chemotherapy and radiation therapy involve high doses of anticancer drugs and radiation, which lead to side effects and serious effects on the overall health of patients [3]. Surgery cannot remove all tumor cells from the primary lesion area and may cause tissue defects that are difficult to self-heal [4]. Photothermal therapy (PTT) is an effective treatment that has been widely considered in recent years. It uses the photo-heat conversion of photothermal agents (PTAs) to produce a hyperthermic effect to destroy cancer cells, and has the advantage of less severe side effects [5]. PTAs are the critical factor in PTT, and the choice and use of the PTAs is important for the success of PTT. Generally, PTAs in the near-infrared biowindows (NIR-I: 650–1000 nm and NIR-II: 1000–1700 nm), with the features of weak damage to tissues, low self-absorption and high tissue penetration, are suitable for tumor treatments [6–8]. According to the chemical composition of PTAs, they can be classified into two categories: organic and inorganic nanomaterials [9]. Organic PTAs are widely used in PTT and include small-molecule dyes and polymer-based nanoparticles (NPs). Inorganic nanomaterials such as carbon nanomaterials, gold nanoparticles, transition metal nanomaterials, and black phosphorus have attracted extensive concern in cancer therapy owing to their unique properties and functions, such as high photothermal conversion efficiency (PCE) and remarkable photothermal stability [10–13].

Although various nanomaterials as PTAs exhibit many excellent properties for PTT applications, they also have some drawbacks [14,15]. When these nanomaterials are administered systematically, they predominantly accumulate in the liver or spleen rather than in cancers [16,17]. When injected locally, most of the nanomaterials are susceptible to rapid removal because they are too small to remain in the interstitial spaces of tissues, which greatly limits the possibility of repeated treatment [18,19]. Additionally, after tumor ablation, especially in breast, bone and skin cancers, there are tumor-induced tissue defects in the area that do not heal easily without additional intervention [20].

With the rapid development of scaffolding technology, the use of three-dimensional (3D) porous scaffolds as carriers of PTAs has aroused great interest in cancer therapy and tissue regeneration [21,22]. In these scaffold-based carriers, the PTAs are incorporated into the scaffold to obtain PTA-integrated composite scaffolds, which enables the PTAs to achieve better photothermal effects *in vivo* [23,24]. In addition, this kind of composite scaffold can not only enable the ablation of cancer cells by PTAs but also support the adhesion and proliferation of normal cells to repair tumor-induced tissue defects because of the similarity of the scaffold to the extracellular matrix (ECM) structure [25,26]. Additionally, the functional modification of composite scaffolds can further mimic the ECM and facilitate new tissue regeneration [27–29].

**Figure 1.**
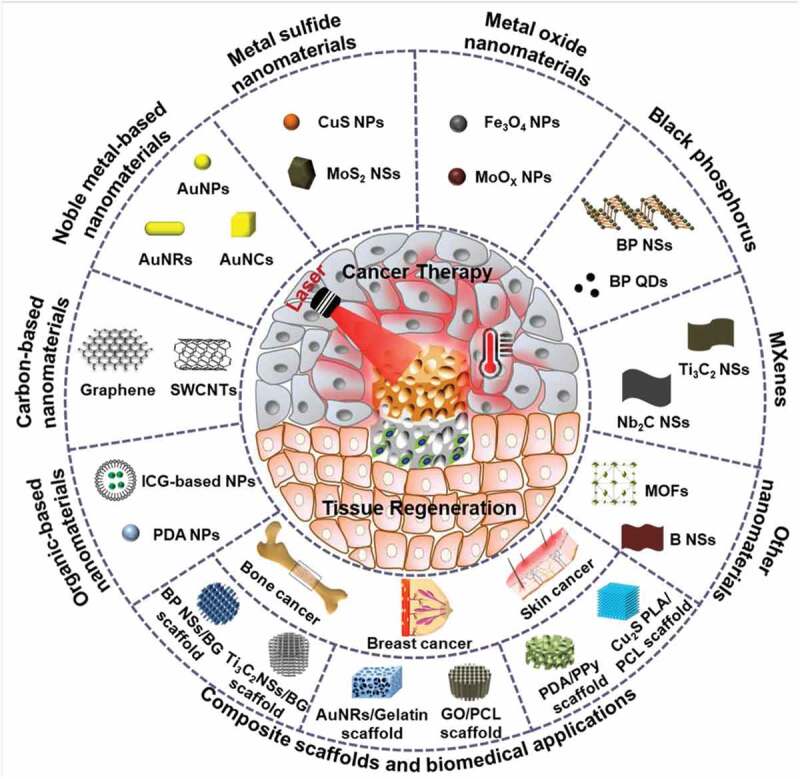
Schematic illustration of various photothermal agents in PTT application and composite scaffolds for integrative cancer therapy and tissue regeneration in bone, skin and breast tissue

This review highlights the latest advances in PTAs for PTT by focusing on organic-based materials, carbon-based materials, metal-based materials, MXenes, and black phosphorus (Figure 1). Further, the integration of various types of nanomaterials with 3D scaffolds for simultaneous cancer therapy and tissue regeneration is summarized. The future prospects and challenges of photothermal nanomaterials and their composite scaffolds are discussed.

## 2. Photothermal agents

A large number of studies have explored the design and preparation of PTAs with high PCE, excellent photostability and good biocompatibility over the past few years [30]. Some typical nanomaterials that are used as PTAs are summarized in Table 1, together with their characteristics and biomedical applications. They can be classified into organic and inorganic PTAs.

**Table 1.** Summary of typical nanomaterials used as photothermal agents for PTT

Type of PTAs	Composition	Size	Irradiation conditions		Cancer cell type	Combined therapy	Application	Ref.
			Wavelength	Power and time				
ICG	ICG@PLGA/ cancer cell membrane	200.4 nm	808 nm	1.0 W cm^−2^, 5 min	MCF-7 cells	PTT	Breast cancer therapy	[36]
PDA	Borate coordination polymer coated PDA/PEGylation DOX	200.0 nm	808 nm	1.5 W cm^−2^, 10 min	MCF-7 cells	PTT/chemotherapy	Breast cancer therapy	[39]
PPy	PPy/PVA	46.0 nm	808 nm	6.0 W cm^−2^, 5 min	HeLa cells	PTT	Cervical cancer therapy	[41]
Semiconducting polymer	PDCDT/PEG	56.0 nm	1064 nm	1.0 W cm^−2^, 10 min	4T1 cells	PTT	Breast cancer therapy	[45]
Graphene	PEGylated Graphene	10–50 nm	808 nm	2.0 W cm^−2^, 5 min	4T1 cells	PTT	Breast cancer therapy	[50]
rGO	PEGylated rGO	18.8 nm	808 nm	15.3 W cm^−2^, 8 min	U87MG cells	PTT	Glioma therapy	[52]
SWCNTs	SWCNT@ mesoporous silica/PEG/DOX	15–25 nm (thickness) 100–300 nm (length)	808 nm	0.5 W cm^−2^, 20 min	4T1 cells	PTT/chemotherapy	Breast cancer therapy	[56]
Gold nanostars	Au nanostars coated BSA/MMP2/IR-780 iodide	121.5 nm	808 nm	0.8 W cm^−2^, 5 min	A549 cells	PTT/PDT	Lung cancer therapy	[75]
Gold nanocages	DOX loaded AuNC@HA	50 nm	808 nm	1.0 W cm^−2^, 5 min	MDA-MB-231 cells	PTT/chemotherapy	Breast cancer therapy	[76]
CuS	Flower-like CuS/PVP	50 nm (thickness) 500–800 nm (length)	980 nm	0.51 W cm^−2^, 10 min	HeLa cells	PTT	Cervical cancer therapy	[84]
MoS_2_	MoS_2_-PEG/DOX	50.0 nm	808 nm	0.56 W cm^−2^, 20 min	4T1 cells	PTT/chemotherapy	Breast cancer therapy	[88]
WS_2_	Gd^3+^/WS_2_/PEG	80.0 nm	808 nm	0.5 W cm^−2^, 10 min	4T1 cells	PTT/radiotherapy	Breast cancer therapy	[91]
Fe_3_O_4_	Perfluorohexane @PLGA/Fe_3_O_4_	347 nm	780–2800 nm	1.5 W cm^−2^, 12 min	SKOV_3_ cells	PTT	Ovarian cancer therapy	[99]
Fe_3_O_4_ nanorings	PEGylated Fe_3_O_4_ nanorings	70 nm (outer diameter)	Magnetic field	30 kA/m, 365 kHz, 10 min	4T1 cells	Magnetic hyperthermia	Breast cancer therapy	[103]
MoO_x_	PEGylated MoO_x_	15–25 nm	1064 nm	0.6 W cm^−2^, 10 min	4T1 cells	PTT/PDT	Breast cancer therapy	[105]
BP nanoparticles	PEGylated BP NPs	3.2 ± 1.0 nm	808 nm	2.0 W cm^−2^, 5 min	4T1 cells	PTT	Breast cancer therapy	[112]
BP nanosheets	DOX loaded PEGylated BP NSs	100 nm	808 nm	1.0 W cm^−2^, 10 min	HeLa cells	PTT/chemotherapy	Cervical cancer therapy	[117]
Ti_3_C_2_	Hyaluronic acid modified Ti_3_C_2_/DOX	100 nm (lateral size)	808 nm	0.8 W cm^−2^, 10 min	HCT 116 cells	PTT/PDT/chemotherapy	Colon cancer therapy	[124]
Zr-FeP MOF	PEGylated siRNA/Zr-FeP MOF	210 nm (length)	635 nm	1.9 W cm^−2^, 5 min	MCF-7 cells	PTT/PDT	Breast cancer therapy	[126]
Boron nanosheets	DOX loaded PEGylated B NSs	250 nm (lateral size) 20 nm (thickness)	808 nm	1.0 W cm^−2^, 10 min	MCF-7 cells	PTT/chemotherapy	Breast cancer therapy	[128]

### 2.1. Organic PTAs

Organic PTAs primarily include small-molecule dyes and polymer-based nanomaterials. Many organic PTAs, such as indocyanine green (ICG) and analogs, and semiconducting polymers, have been used for PTT due to their good biocompatibility, high PCE, and simple synthesis. They are summarized and discussed in the categories of organic small molecule-based and polymer-based PTAs.

#### 2.1.1. Organic small molecule-based PTAs

Cyanine-based dyes (ICG and cypate) are typical organic small-molecule PTAs. ICG that was approved by FDA for clinical ophthalmic angiography in 1959 (NDA#011525) is one of the most popular NIR dyes for PTT applications [31]. Chen et al. did a series of pioneering studies that investigated ICG as a PTA delivered intratumorally for photothermal ablation [32–34]. They also reported the enhanced efficacy of immunotherapy after ICG photothermal ablation in metastatic breast cancer [35]. ICG by intratumoral injection could directly ablate primary tumors under laser irradiation and induce immunoadjuvant-directed stimulation for immune responses to eradicate untreated metastases at remote sites.

To further improve the targeting capacity and prolong blood circulation time, ICG has been modified with biocompatible components. Chen et al. integrated ICG with tumor cell membrane to form core-shell nanoparticles [36]. The prepared nanoparticles demonstrated specific targeting to tumor cells and good photothermal responsiveness, resulting in efficient thermal ablation of xenograft tumors under 808 nm laser irradiation.

#### 2.1.2. Organic polymer-based PTAs

Some semiconducting polymers, such as polydopamine (PDA), and polypyrrole (PPy), exhibit light absorption in the NIR region that makes them useful for PTT. In addition, because these polymers exhibit higher photostability, better biocompatibility, and longer half-life in blood than the organic small-molecule dyes, they have attracted extensive attention as PTAs [37,38]. Liu et al. developed a nanoplatform for PTT combined with chemotherapy using PEGylated borate coordination polymer (CP)-coated PDA NPs (PDA/CP-PEG/DOX) [39]. The photothermal effect of PDA and the chemotherapeutic effect of the porous CP layer loaded with doxorubicin (DOX) provide a potential synergistic therapeutic effect. PEGylation could attenuate NPs elimination by the reticuloendothelial system and increase passive targeting ability. The PDA/CP-PEG/DOX showed low system toxicity, effective tumor targeting and good chemo-photothermal tumor suppressive activity. PPy, because of its good biocompatibility and stability, can be an outstanding candidate for PTT application [40]. Zha et al. synthesized uniform PPy nanoparticles via a one-step aqueous dispersion polymerization strategy [41]. The as-prepared PPy nanoparticles exhibited good photostability and PCE and could effectively kill HeLa cells by the hyperthermic effect of PPy nanoparticles. Zhang et al. prepared PPy nanoparticles by a microwave-assisted method, and the synthesis time was shortened to 2 minutes [42]. The obtained PPy nanoparticles possessed uniform size, excellent water solubility, and good photothermal performance. An *in vitro* photothermal experiment using the PPy nanoparticles led to significant death of cancer cells, and *in vivo* tumor ablation under 808 nm laser irradiation was achieved.

Semiconducting polymer PTAs are composed of a highly extended π-conjugated main chain with two alternating parts, an electron donor and an electron acceptor. The band gap between the highest occupied molecular orbital and the lowest unoccupied molecular orbital can be easily tuned for strong NIR absorption, good light stability, and ideal biophysical properties [43,44]. Jiang et al. prepared a photothermal semiconducting polymer nanopartciles with two simultaneous absorption peaks in the NIR-I and NIR-II biowindows [45]. The nanoparticles had a PCE of 44.9 and 43.4% at 808 nm and 1064 nm, respectively. Its deep-tissue photoheating capabilities under NIR-I and NIR-II light were compared. When the tissue depth was 2, 5, 10 and 20 mm, the maximum photothermal temperature of the nanoparticles irradiated at 1064 nm was 4.6, 4.7, 8.5 and 3.3 times higher than that at 808 nm, respectively. *In vivo* and *in vitro* investigation showed that the nanoparicles could effectively ablate cancer cells without damage to normal tissues under 1064 nm laser irradiation.

### 2.2. Inorganic PTAs

Since Hirsch et al. first introduced metal nanoshells for the PTT of human breast carcinoma tumors in 2003 [46], various inorganic nanomaterials have been prepared and used as PTAs for PTT applications. The primary inorganic PTAs include carbon-based nanomaterials, noble metal-based nanomaterials, metal sulfide nanomaterials, metal oxide nanomaterials, MXenes, and black phosphorus nanosheets. The latest advances in PTT based on these inorganic materials are summarized and compared.

#### 2.2.1. Carbon-based nanomaterials

Carbon-based nanomaterials, such as graphene, graphene oxide (GO), reduced graphene oxide (rGO), and carbon nanotubes (CNTs), have distinctive chemical and physical properties and have been extensively used in cancer treatment [47,48].

Graphene is composed of one or several single-atom-thick layers of sp^2^-bonded carbon atoms. Graphene exhibits excellent physical characteristics, such as plasma properties, which enables graphene and its derivatives to transform light energy into heat energy through the photothermal effect of plasma. When graphene materials are irradiated by light, surface plasmons can be activated to generate random dipoles and resonance and are finally converted into heat energy, which is the main reason graphene-based materials can perform PTT [49]. Yang et al. first studied the *in vivo* behavior of PEGylated GO (GO-PEG) in tumor bearing mice and found that GO-PEG showed high efficiency in passive tumor targeting and low retention in reticuloendothelial systems [50]. Moreover, due to the strong NIR absorption of GO-PEG, effective tumor ablation was achieved *in vivo* through the intravenous injection of GO-PEG and 808 nm laser irradiation at a safe power level. Chang et al. took advantage of the low toxicity and high specific surface area of GO to develop a versatile GO/BaHoF_5_/PEG nanocomposite. The as-prepared GO nanocomposite showed good biocompatibility and achieved effective ablation of cancer cells under a safe power density *in vivo* [51]. rGO is a well-known graphene derivative. Robinson et al. prepared PEG-modified rGO with good aqueous solubility and high-efficiency photothermal ablation under 808 nm laser irradiation [52]. Compared with PEGylated GO, the NIR absorption of the PEGylated rGO was enhanced by 6 times under the same conditions.

As a type of carbon nanomaterial, single-walled carbon nanotubes (SWCNTs), which are composed of sp^2^ carbon sheets, also have excellent light absorption characteristics. Lu et al. reported the low toxicity of SWCNTs bound with targeting antibodies [53]. *In vivo* and *in vitro* studies showed that the SWCNTs showed stable photothermal properties and good biosafety and could achieve precise targeting of tumors for photothermal ablation. In addition, by bonding or wrapping, CNTs can be easily functionalized with different medical molecules or nanomaterials such as drugs, biomolecules and magnetic NPs [54,55]. In this way, the photothermal performance of CNTs can be improved, and synergetic therapy can be developed. For instance, Liu et al. fabricated a multifunctional nanoplatform by the PEGylation of mesoporous silica (MS) coated SWCNTs, which were used as a carrier of anticancer drugs and a PTA for combination therapy of cancer [56]. SWCNT/MS-PEG could efficiently load doxorubicin (DOX) in the mesoporous structure of SWCNTs. The SWCNT/MS-PEG/DOX exhibited NIR light-dependent drug release behavior, which achieved an outstanding tumor synergetic inhibition effect in an animal tumor model.

The carbon-based nanomaterials with high NIR absorbance and excellent PCE have been used as PTA for many years. However, the potential long-term toxicity of carbon-based nanomaterials is a major obstacle to their future clinical application.

#### 2.2.2. Noble metal-based nanomaterials

Noble metal-based nanomaterials are one of the most explored nanomaterials for PTT [57–59], and are prepared from Au, Ag, Pt, and Pd [60,61]. The PTT of noble-metal nanomaterials is based on their optical property of localized surface plasmon resonance (LSPR), which is a collective oscillation of light-induced free electrons. By changing the size, shape, ligand, and composition of the nanoparticles, the wavelength of LSPR can be adjusted in the NIR region [62]. Thus, various sizes and shapes of noble-metal nanomaterials, including nanorods, nanospheres, nanocages, nanostars, and nanosheets, have been studied for enhancing PTT properties [63–65].

Gold nanoparticles show typical absorption bands in the 500 to 550 nm region, and increasing the size does not allow the absorption peak to be fine-tuned in the NIR region, which limits its application in PTT [66]. However, when the interparticle gap decreases, the absorption peak of the LSPR of AuNPs can be shifted from visible light to the NIR region. Therefore, gold nanospheres with a few nanometers are often used to construct gold-nanoaggregates for PTT applications in cancers [67,68]. Park et al. synthesized albumin-containing gold-nanoaggregates (~88 nm) by mixing the gold nanospheres (~4.5 nm) with albumin and promoting their agglomeration [69]. The as-prepared gold-nanoaggregates exhibited stronger absorption in the wavelength of 600–900 nm and elevated the tumor temperature of colon tumor-bearing mice over 50°C *in vivo* under 808 nm laser irradiation, which markedly suppressed cancer growth.

Gold nanorods (AuNRs) are among the most interesting morphologies developed for PTT applications. Depending on the diameter and length of the rods, AuNRs have two characteristic optical absorptions, namely, transverse and longitudinal optical absorption, respectively. Therefore, by adjusting the length/diameter ratio, the LSPR of AuNRs can be shifted to the NIR region, and the PCE of AuNRs can be significantly improved for PTT [70]. Mackey et al. determined optimal AuNR size by comparing the plasma characteristics of three different sizes of AuNRs [71]. Among three different AuNRs with an aspect ratio of 3.4–3.5 (38 × 11, 28 × 8, and 17 × 5 nm), they found that the 28 × 8 nm AuNRs were the most effective in plasma photothermal generation and in thermal ablation of HSC-3 tumor cells. In addition, because of simple surface modification, AuNRs are attractive nanocarriers for loading drugs, antibodies, photosensitizers and genes, which paves the way for synergistic cancer therapy, such as PTT/chemotherapy [72], PTT/PDT [73], PTT/gene therapy [74].

**Figure 2.**
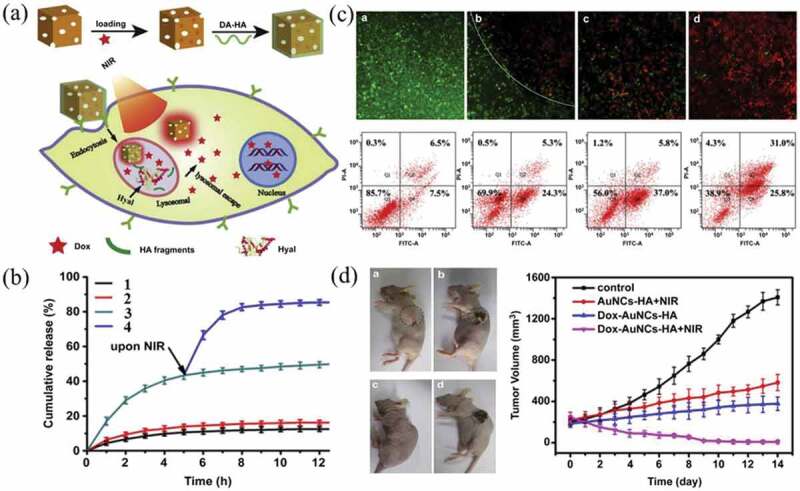
(a) Schematic representation of stimuli-responsive DOX-loaded AuNC/HA nanoparticles for pinpointed intracellular drug release and synergistic therapy. (b) The DOX release profiles from DOX/AuNCs/HA in acetate buffer at pH 4.5: (1) control, (2) upon NIR irradiation, (3) in the presence of hyaluronidase, (4) in the presence of Hyal upon NIR irradiation. (c) Live/dead and flow cytometry apoptosis assay. (d) The relative tumor volumes of nude mice versus time after treatment. Control (a), AuNCs/HA upon NIR irradiation (b), DOX/AuNCs/HA (c), DOX/AuNCs/HA upon NIR irradiation (d). Reproduced with permission [76]

Gold nanostars and nanocages have been extensively investigated for PTT. Xia et al. reported a simple strategy to synthesize gold nanostars coated with matrix metalloproteinases (MMP2) polypeptides and IR-780 iodide through bovine serum albumin (BSA) for targeted imaging and enhanced PTT/PDT in lung cancer [75]. Wang et al. reported a multi-stimuli-responsive platform based on drug-loaded gold nanocages (AuNCs) @hyaluronic acid (DOX/AuNCs/HA) (Figure 2(a))[76]. DOX could be released from DOX/AuNCs/HA in intracellular environments, and the release was accelerated upon 808 nm laser irradiation (Figure 2(b)). Both *in vitro* and *in vivo*, the DOX/AuNCs/HA exhibited strong anti-cancer effect (Figure 2(c, d)), which dramatically improved the therapeutic efficacy compared to chemotherapy or photothermal treatment alone.

In addition to Au nanomaterials of various shapes, Ag nanomaterials have been used in PTT of tumors due to the LSPR properties and the toxicity of Ag^+^ [77,78]. Similarly, Pt nanomaterials have also been reported to generate enough heat to ablate tumors under NIR laser irradiation [79,80].

#### 2.2.3. Metal sulfide nanomaterials

Metal sulfide nanomaterials, which are lower in cost than noble metals, are also widely used in PTT. The CuS and copper-deficient structures (Cu_2-x_S) exhibit an NIR absorption due to the d-d energy band transition of Cu^2+^ ions, which allows them to be used as PTAs [81,82]. Zhou et al. developed both citrate- and polyethylene glycol (PEG)-stabilized CuS NPs, which displayed a strong absorption at 930 nm [83]. The CuS NPs showed a passive targeting effect *in vivo* and could promote the photothermal ablation of U87 tumor cells under exposure to 808 nm laser. The size and shape of copper chalcogenide nanomaterials play a prominent role in the photothermal performance. Tian et al. reported hydrophilic flower-like CuS superstructures as an efficient 980 nm laser-driven PTA for the photothermal ablation of tumors [84]. The flower-like CuS exhibited high PCE that was increased by approximately 50% compared to that of its corresponding architectural hexagonal nanoplates and could efficiently ablate cancer cells by its photothermal effect under 980 nm laser irradiation. Wang et al. reported that PEG-coated NIR plasmonic Cu_2-x_S nanocrystals showed dual PTT and PDT cytotoxic effects on melanoma [85].

MoS_2_, a representative transition-metal sulfide, is of great interest in PTT of cancers [86]. Chou et al. synthesized chemically exfoliated MoS_2_ nanosheets as NIR PTAs [87]. MoS_2_ nanosheets displayed approximately 7.8 times stronger absorption in the NIR region than GO with an extinction coefficient of 29.2 L g^−1^ cm^−1^ at 800 nm. Liu et al. developed multifunctional MoS_2_ nanosheets as a drug carrier and a PTA for PTT combined with chemotherapy to treat cancer [88]. MoS_2_ nanosheets were synthesized by chemical exfoliation and then modified with lipoic acid-modified PEG (LA-PEG) to improve their physiological stability and biocompatibility. Both *in vitro* and *in vivo* studies showed that MoS_2_-PEG nanosheets had no obvious cytotoxicity. Furthermore, the DOX-loaded MoS_2_-PEG nanosheets, combined with PTT, had excellent synergistic anticancer effects in suppressing breast tumor growth *in vivo*.

Recently, metal sulfide nanomaterials such as WS_2_ and Bi_2_S_3_, have also been reported as promising PTAs for cancer treatment owing to their strong absorbance in the NIR region [89,90]. Cheng et al. reported a general strategy to dope Gd^3+^ ions in WS_2_ nanoflakes modified with PEG as diagnostic and therapeutic agents (Gd^3+^/WS_2_/PEG) for imaging-guided PTT/radiation therapy of cancers [91].

These studies indicate that metal sulfide nanomaterials have distinct features, such as high NIR absorbance, tunable components, and favorable photothermal stability. In addition, metal sulfide nanomaterials can integrate with other therapeutic molecules to achieve a combination of PTT, PDT, chemotherapy and radiation therapy. Although the metal sulfide nanomaterials have aroused great research interest, there is still a long way to go in clinical applications.

#### 2.2.4. Metal oxide nanomaterials

In addition to the metal sulfide nanomaterials, metal oxide nanomaterials, such as magnetic iron oxide, molybdenum oxide and manganese oxide, have been proven to be highly popular PTAs for PTT [92–94]. Iron oxide nanomaterials have been extensively studied to produce a thermal response under NIR light radiation and the presence of alternating magnetic fields [95]. Iron oxides include γ-Fe_2_O_3_, Fe_3_O_4_ and FeO, among which the most studied are γ-Fe_2_O_3_ and Fe_3_O_4_ because they exhibit superparamagnetism, high specific surface area, and biocompatibility [96]. Furthermore, iron oxide nanoparticles were approved by FDA in 2009 as iron replacement in the treatment of iron deficiency anemia in patients with chronic kidney disease (NDA#022180). In general, iron oxide nanomaterials can be synthesized by many methods, such as co-precipitation, thermal decomposition, or hydrothermal or solvothermal synthesis [97,98]. Zhao et al. prepared nanocapsules with shells of a mixture of poly(lactic-co-glycolic acid) (PLGA) and superparamagnetic iron oxide (Fe_3_O_4_) and cores of perfluorohexane (PFH) as diagnostic and therapeutic agents (PFH@PLGA/Fe_3_O_4_) for imaging-guided PTT [99]. After exposure to NIR laser irradiation, the thermal effect of infrared radiation and the thermoelastic expansion effect of the phase transition of PFH@PLGA/Fe_3_O_4_ synergistically caused agglutinative necrosis and damage of cancer cells. Moreover, the Fe_3_O_4_ NPs and PFH could serve as favorable contrast agents for magnetic resonance (MR) and ultrasound imaging, respectively. Yang et al. reported hyaluronic acid (HA)-functionalized superparamagnetic iron oxide nanoparticles (HA-SPIONs) for T2-weighted MR imaging and PTT of breast cancer with CD44 HA receptor overexpression [100].

**Figure 3.**
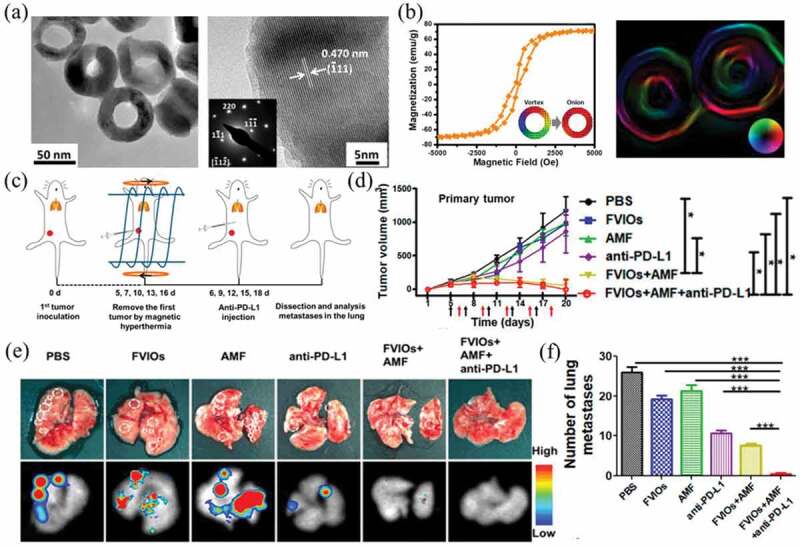
(a) TEM and HRTEM images of PEGylated FVIOs. (b) Magnetic hysteresis loop and Lorentz TEM image of PEGylated FVIOs. (c) Schematic illustration of animal experimental design. (d) Tumor volume versus time after treatment. (e) Representative photographs of lungs and bioluminescence images after treatment. (f) Number of tumor nodules present in the lungs. Reproduced with permission [103]

Recently, due to their special magnetic response, magnetic iron oxide nanoparticles have attracted widespread attention for magnetic hyperthermia treatment (MHT) under an alternating magnetic field to selectively ablate cancer cells [101,102]. Liu et al. constructed PEGylated ferrimagnetic vortex-domain iron oxide nanorings (FVIOs-PEG) (Figure 3(a)), which could achieve a stable vortex domain and excellent magnetocaloric performance (Figure 3(b)) [103]. The biocompatible FVIOs-PEG generated mild heat under an alternating magnetic field, which induced apoptosis and calreticulin (CRT) exposure on the surface of 4T1 cells. In an orthotopic 4T1 tumor model, the combination of FVIOs-PEG magnetic hyperthermia and anti-PD-L1 therapy not only eliminated the primary tumors treated with an alternating magnetic field locally but also significantly prevented lung metastasis (Figure 3(c-f)).

Other metal oxides such as MoO_x_ and MnO_x_ can also be used as PTAs in antitumor PTT. Liu et al. synthesized a new type of bow tie-like MoO_2_ NPs with strong LSPR effect, high PCE, and low toxicity [104]. *In vitro* and *in vivo* studies indicated that the MoO_2_ NPs produced a very impressive thermal ablation effect on cancer cells. Yin et al. reported plasmonic PEGylated MoO_x_ NPs (MoO_x_-PEG) by using a facile hydrothermal method [105]. The MoO_x_-PEG exhibited strong absorption and marked PCE in both the 808 nm and 1064 nm NIR biowindows. The photothermal ablation effects of PEG-MoO_x_ for tumor cells were achieved under 808 nm and 1064 nm laser irradiation, and these NPs could be gradually eliminated by metabolism from the liver and spleen of mice. In addition, the MoO_x_-PEG exposed to 1064 nm laser irradiation could not only effectively convert light into heat but also stimulate the formation of ROS, which induced dramatic tumor cell death *in vivo* due to the synergistic PTT/PDT effect.

#### 2.2.5. Black phosphorus

**Figure 4.**
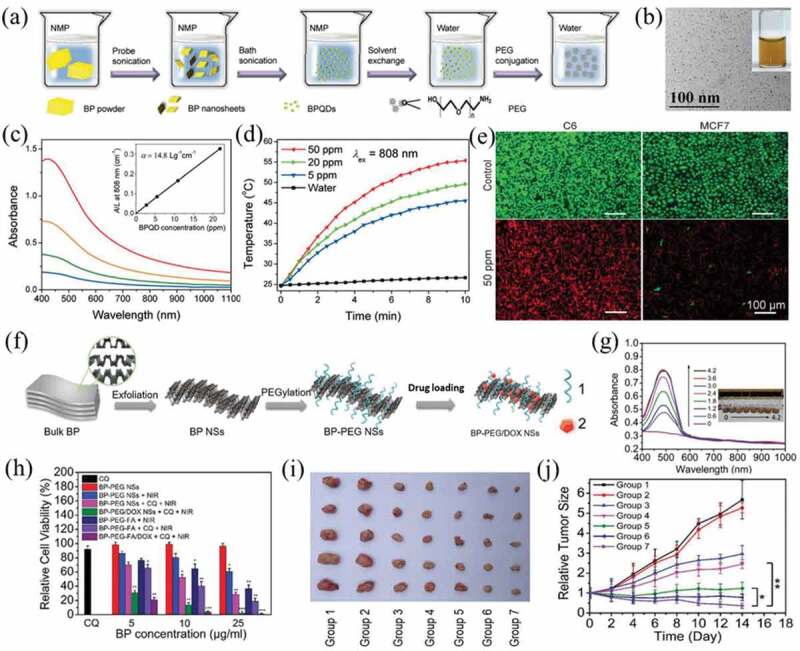
(a) Synthesis and surface modification BPQDs. (b) TEM image and photographs of BPQDs in water. (c) Absorbance spectra of BPQDs dispersed in water at different concentrations. (d) Photothermal heating curves of pure water and BPQDs dispersed in water at different concentrations. (e) Live/dead staining of C6 and MCF7 cancer cells. Reproduced with permission [111]. (f) Schematic representation of the PEGylated BP theranostic delivery platform. 1: PEG-NH_2_, 2: DOX. (g) Absorbance spectra of BP-PEG/DOX NSs at different concentrations. (h) Relative viability of HeLa cells after different types of treatment with different BP concentrations. (i) Photographs of tumors and (j) inhibition of tumor growth after different treatments. Group 1: saline; Group 2: chloroquine (CQ); Group 3: DOX; Group 4: BP-PEG-FA/DOX; Group 5: BP-PEG-FA+NIR; Group 6: BP-PEG-FA+NIR+CQ; Group 7: BP-PEG-FA/DOX+NIR+CQ. Reproduced with permission [117]

Since the first successful exfoliation in 2014, black phosphorus (BP) has attracted widespread attention for its unique structure and properties [106]. Due to the weak van der Waals forces between stacked BP layers, BP can be easily peeled off into a single layer or several layers of nanosheets. The wide tuning range of the band gap in BP allows wide absorption in the NIR region, so it can be used for PTT, PDT and PA imaging [107,108]. In addition, BP exhibits relatively low cytotoxicity and good biocompatibility because BP is easily biodegradable in the body and can produce nontoxic intermediates such as phosphate, phosphite and other P_x_O_y_ upon coming into contact with water and oxygen, which also further increases its clinical application prospects [109,110]. Sun et al. synthesized ultrasmall BP quantum dots (BPQDs) by using a liquid exfoliation strategy that incorporated probe and bath sonication (Figure 4(a)) [111]. The ultrasmall BPQDs that had a lateral size of approximately 2.6 nm and a thickness of approximately 1.5 nm exhibited excellent photothermal performance and photostability with a high extinction coefficient of 14.8 L g^−1^ cm^−1^ and a PCE of 28.4% at 808 nm (Figure 4(b-d)). After PEG modification, the BPQDs-PEG showed enhanced stability in physiological medium, and its toxicity to cells was basically minimal. Further *in vitro* studies demonstrated a high PTT efficiency of the BPQDs-PEG in inducing cancer cell death (Figure 4(e)). Sun et al. reported water-soluble and biocompatible PEGylated BPQDs that were prepared by one-pot solventless high energy mechanical milling technique [112]. *In vitro* experiments confirmed the excellent biocompatibility of PEGylated BPQDs and the photothermal ablation of cancer cells under 808 nm laser irradiation. *In vivo* experiments showed that the PEGylated BPQDs could serve as a PTA for the PA imaging-guided PTT of cancer.

In addition to BPQDs, BP nanosheets (BPNSs) have made some progress in cancer treatment [113]. Fu et al. prepared three kinds of BPNSs with different sizes (namely, large BP (394 nm ± 75 nm), medium BP (118 nm ± 22 nm) and small BP (4.5 nm ± 0.6 nm)) by using an ameliorated liquid exfoliation technique [114]. Three different sizes of BPNSs were compared for their cytocompatibility and photothermal effect. All the three types of BPNSs possessed excellent biocompatibility, and the larger BPNSs showed better thermal ablation of cancer cells.

BPNSs can also be used as nanocarriers by taking advantage of their large surface area to deliver chemotherapy drugs, genes and biomolecules [115,116]. Tao et al. designed a theranostic delivery platform based on BPNSs for cancer therapy (Figure 4(f)) [117]. BPNSs were prepared from bulk BP by a mechanical exfoliation strategy and then modified with positively charged PEG-NH_2_ by electrostatic adsorption to improve their biocompatibility and physiological stability (Figure 4(g)). The PEGylated BPNSs were loaded with doxorubicin (DOX) and folate for chemotherapy, and the as-prepared DOX-loaded PEGylated BPNSs exhibited enhanced antitumor effects both *in vitro* and *in vivo* (Figure 4(h-j)).

#### 2.2.6. MXenes

MXenes are a class of multifunctional 2D nanomaterials including transition metal carbides and carbonitrides with many attractive features [118,119]. The general formula of an MXene is M_n+1_X_n_, where M represents an early transition metal, and X represents carbon or nitrogen, n = 1, 2, 3 [120]. MXenes possess excellent absorption in the NIR range and high PCE, demonstrating broad application prospects. Ti_3_C_2_ nanosheets were the first MXenes that were proven to be an effective PTA for PTT. Xuan et al. utilized organic base-driven intercalation and delamination to produce modified Ti_3_C_2_ nanosheets on the basis of the most widely studied Ti_3_AlC_2_ [121]. The as-prepared Ti_3_C_2_ nanosheets manifested strong absorption in the NIR region and exhibited a high extinction coefficient of 29.1 L g^−1^ cm^−1^ at 808 nm. Next, they used the Ti_3_C_2_ nanosheets as a PTA for 4T1 cell ablation under 808 nm laser irradiation and showed that these nanosheets could effectively ablate cancer cells. Lin et al. also synthesized Ti_3_C_2_ nanosheets modified with soybean phospholipid (Ti_3_C_2_-SP) based on two-step exfoliation of MAX (M is an early transition metal carbide, A is an A group element, and X is C or N.) phase Ti_3_AlC_2_ [122]. The Ti_3_C_2_-SP nanosheets demonstrated highly effective hyperthermia against cancer cells through intravenous or intratumor injection of the Ti_3_C_2_-SP nanosheets and 808 nm laser irradiation.

Ta_4_C_3_ nanosheets, another type of MXene, have also been reported for use in PTT. Lin et al. reported Ta_4_C_3_ nanosheets with nanosized lateral widths obtained by the two-step liquid exfoliation of MAX phase Ta_4_AlC_3_ [123]. Compared to Ti_3_C_2_ nanosheets, these Ta_4_C_3_ nanosheets possessed a biocompatible Ta component and higher PCE of 44.7% in addition to excellent photothermal stability. Further *in vivo* photothermal therapeutic experiments with Ta_4_C_3_ nanosheets under laser irradiation resulted in very severe necrosis of cancer cells and negligible cytotoxicity to normal cells.

The 2D MXenes have a large surface area that allows easy loading of various therapeutic agents, such as chemotherapeutics and biomacromolecules. Liu et al. synthesized Ti_3_C_2_ nanosheets by a surface modification method [124]. The Ti_3_C_2_ nanosheets exhibited a high extinction coefficient of 28.6 L g^−1^ cm^−1^ and a PCE of 58.3% at 808 nm. Furthermore, through the layer-by-layer adsorption of doxorubicin (DOX) and tumor-targeted HA, a multifunctional nanoplatform based on Ti_3_C_2_ nanosheets (Ti_3_C_2_/DOX/HA) with a drug loading capacity of up to 84.2% was achieved. The Ti_3_C_2_/DOX/HA displayed tumor site-specific accumulation, controlled stimuli-responsive drug release, and synergistic cancer therapy effects *in vivo*.

#### 2.2.7. Other nanomaterials

In addition to the nanomaterials mentioned above, some other nanomaterials such as metal-organic frameworks (MOFs) have also been explored as PTAs for PTT applications. MOFs are a class of molecular crystalline materials that consist of metal ions or clusters bridged by organic linkers [125]. Zhang et al. prepared zirconium-ferriporphyrin MOF (Zr-FeP) nanoshuttles by a simple one-pot hydrothermal strategy and then loaded heat shock protein 70 (Hsp70) inhibitor siRNA on the nanoshuttles to obtain siRNA/Zr-FeP [126]. Under NIR laser irradiation, the siRNA/Zr-FeP enabled the simultaneous generation of heat and reactive oxygen species (ROS), in which Hsp70 siRNA could improve the mild temperature PTT. Both *in vitro* and *in vivo* experiments showed that the siRNA/Zr-FeP could combine PDT with mild temperature PTT, resulting in significant suppression of cancer growth.

Nanocomposites can integrate various nanomaterials to conquer the drawbacks of individual nanomaterials, such as poor biocompatibility and PCE. Yang et al. designed a novel nanocomposite by the assembly of Fe_3_O_4_ nanoparticles and Au nanoparticles on BP nanosheets (BPs@Au@Fe_3_O_4_), which exhibited a strong NIR absorption [127]. *In vitro* and *in vivo* studies indicated that the BPs@Au@Fe_3_O_4_ NPs showed high biocompatibility and excellent anticancer efficacy owing to a synergistic PTT/PDT effect mediated by low laser power. Moreover, the BPs@Au@Fe_3_O_4_ could visualize the tumor growth *in vivo* with the help of MR imaging.

Ultrathin boron (B) nanosheets are another interesting 2D nanomaterial developed as PTAs for PTT. Ji et al. developed a new photonic drug delivery platform based on the PEGylated B NSs [128]. The as-prepared B NSs exhibited multiple promising features: a high PCE of 42.5%, high DOX-loading capacity and release triggered by NIR laser irradiation, excellent biocompatibility, and strong accumulation at tumor sites. *In vitro* and *in vivo* experiments demonstrated the outstanding synergistic anticancer effect of the DOX-loaded PEGylated B NSs.

## 3. 3D scaffolds for cancer therapy and tissue regeneration

Cells are surrounded by their 3D extracellular matrices (ECMs) *in vivo*. Nanomaterials need to pass through the ECM to reach the cells. In recent years, PTAs have been incorporated into 3D scaffolds to construct composite scaffolds for efficient delivery to cancer cells [129,130]. The composite scaffolds can be implanted in targeting sites for the photothermal ablation of cancer cells or possible residual tumor cells after surgery. Local implantation of the composite scaffolds can constrain the localization of PTAs in the implanted sites, and therefore the composites can be repeated irradiated for the repeated ablation of cancer cells. The composite scaffolds can be designed for multiple functions. At the initial stage after implantation, the composite scaffolds act as PTAs for PTT ablation of cancer cells. At the late stage after cancer eradication, tissue reconstruction and regeneration are required for large defects. The 3D composite scaffolds can serve as templates to provide necessary spaces and microenvironments for the regeneration of new tissues and organs. The composite scaffolds can stimulate the migration, adhesion, proliferation, and differentiation of cells, such as mesenchymal stem cells (MSCs) [131], and further promote the healing of tissue defects induced by tumor thermal ablation or resection, especially in breast, bone or skin cancer. Some typical composite scaffolds of photothermal nanomaterials and their preparation methods, properties and biomedical applications are summarized in Table 2. Details of these composite scaffolds are introduced and discussed in the following sections.

**Table 2.** Summary of composite scaffolds of photothermal nanomaterials used for cancer therapy and tissue regeneration applications

Type of PTAs	Scaffold	Fabrication method	Irradiation conditions		Cell type		Application	Ref.
			Wavelength	Power and time	Cancer	Normal tissue		
PDA	PDA modified alginate scaffold	3D printing method	808 nm	0.5 W cm^−2^, 5 min	4T1 cells	MCF-10A cells	Breast cancer therapy and breast reconstruction	[132]
PPy, PDA	PPy@PDA hydrogel scaffold	Freeze-drying method	808 nm	1.41 W cm^−2^, 10 min	A375 cells	L929 cells	Skin cancer therapy and wound healing	[136]
OPC	OPC containing hydrogel scaffold	3D printing method	808 nm	1.2 W cm^−2^, 15 min	B16F10 cells	HUVECs/HDFs	Skin cancer therapy and wound healing	[137]
Carbon dots	CD doped chitosan/ nanohydroxyapatite scaffold	Freeze-drying method	808 nm	1.0 W cm^−2^, 10 min	UMR-106 cells	rBMSCs	Bone cancer therapy and reconstruction	[140]
Graphene oxide	GO/polycaprolactone scaffold	Salt template assisted method	808 nm	0.75 W cm^−2^, 1 min	MCF-7 cells	ADSCs	Breast cancer therapy and adipose tissue repair	[141]
Graphene oxide	PCL microfiber/GO scaffold	Electrospinning method	810 nm	10 W cm^−2^, 200 s	MCF-7 cells	HDFs	Breast cancer therapy and adipose tissue repair	[142]
Au nanostars/rods	Au nanostars/rods integrated Gelatin scaffold	Ice particulate templating method	805 nm	1.6 W cm^−2^, 3.0 min	HeLa cells	/	Cervical cancer therapy	[143]
Au nanorods	AuNRs integrated Gelatin scaffold	Ice particulate templating method	805 nm	1.6 W cm^−2^, 6.0 min	4T1 cells	hMSCs	Breast cancer therapy and adipose tissue repair	[146]
Au nanocages	AuNCs integrated BCP scaffold	Sintering method	690/808 nm	1.0 W cm^−2^, 10 min	/	Macrophages/Dendritic cells	Bone regeneration	[147]
Cu_2_S	Cu_2_S incorporated PLA/PCL scaffold	Electrospinning method	808 nm	0.4 W cm^−2^, 15 min	B16F10 cells	HUVECs/HDFs	Skin cancer therapy and wound healing	[149]
CuFeSe_2_	CuFeSe_2_ integrated BG scaffold	3D printing method	808 nm	0.55 W cm^−2^, 10 min	Saos-2 cells	rBMSCs	Bone cancer therapy and reconstruction	[150]
MoS_2_	MoS_2_ modified Akermanite scaffold	3D printing method	808 nm	0.5 W cm^−2^, 10 min	Saos-2 cells	rBMSCs	Bone cancer therapy and reconstruction	[152]
Fe_3_O_4_	Gelatin/Fe_3_O_4_ composite scaffold	Ice particulate templating method	805 nm	1.6 W cm^−2^, 3.0 min	HeLa cells	/	Cervical cancer therapy	[157]
Fe_3_O_4_	Fe_3_O_4_/GdPO_4_/CS scaffold	Freeze-drying method	808 nm	4.6 W cm^−2^, 150 s	MDA-MB-231 Cells	hBMSCs	Breast cancer-induced bone metastases therapy and reconstruction	[158]
Fe_3_O_4_	Fe_3_O_4_/GO composite scaffold	3D printing method	Magnetic field	180 Gs/409 kHz, 20 min	MG63 cells	rBMSCs	Bone cancer therapy and reconstruction	[160]
BP nanosheets	BP NSs integrated BG scaffold	3D printing method	808 nm	1.0 W cm^−2^, 5 min	Saos-2 cells	hBMSCs	Bone cancer therapy and reconstruction	[162]
BP nanosheets	BP NSs incorporated Gelatin-PCL scaffold	Electrospinning method	808 nm	0.65 W cm^−2^, 15 min	B16F10 cells	HUVECs/NIH-3T3 cells	Skin cancer therapy and wound healing	[163]
Nb_2_C	Mesoporous Silica@Nb_2_C/BG scaffold	3D printing method	1064 nm	1.0 W cm^−2^, 10 min	Saos-2 cells	hBMSCs	Bone cancer therapy and reconstruction	[167]
Ti_3_C_2_	Ti_3_C_2_/BG scaffold	3D printing method	808 nm	1.0 W cm^−2^, 10 min	Saos-2 cells	hBMSCs	Bone cancer therapy and reconstruction	[169]
Cu-TCPP	Cu-TCPP/TCP scaffold	3D printing method	808 nm	0.9 W cm^−2^, 10 min	Saos-2 cells	hBMSCs/HUVECs	Bone cancer therapy and reconstruction	[170]
Bi_2_O_3_	Bi_2_O_3_ doped BG scaffold	Melting and quenching technique	808 nm	1.5 W cm^−2^, 10 min	UMR106 cells	MC3T3-E1 cells	Bone cancer therapy and reconstruction	[171]

### 3.1. Scaffolds incorporating organic PTAs

In most cancer treatments, tissue defects and residual tumor cells are two key issues for prognosis. Therefore, in the treatment process, it is important to create multifunctional scaffolds that can both kill the cancer cells and repair the tissues. In recent research, a series of strategies have been proposed for combining PTAs with engineering scaffold materials, such as hydrogels, electrospun nanofibers, and 3D printed scaffolds, to repair tissue defects after the ablation of tumor tissue. Organic PTAs have been hybridized with alginate, silk fibroin (SF) and bioactive glass (BG) to prepare PTT composite scaffolds [132–134].

Alginate has good biocompatibility and exhibits controlled gelation, and it is therefore widely used in soft tissue regeneration [135]. Luo et al. integrated PDA and alginate into a dual-functional scaffold by 3D printing [132]. The alginate-polydopamine (PDA/Alg) composite scaffold exhibited good photothermal ablation of 4T1 cancer cells *in vivo* under 808 nm laser irradiation, which efficiently prevented the local recurrence of breast cancer. In addition, the PDA/Alg composite scaffolds possessed high elasticity and modulus values similar to those of normal breast tissues and could promote the adhesion and proliferation of normal breast epithelial MCF-10A cells. Miao et al. reported the use of PDA-modified SF composite scaffolds to achieve effective bone cancer treatment [133]. PDA/SF composite scaffolds were prepared by a freeze-drying method and had micropores with a size of 200–250 µm that could accommodate cells in the tissue regeneration process. The PDA/SF composite scaffolds showed excellent photothermal effects, inducing significant cytotoxicity to human osteosarcoma cells under 808 nm laser irradiation. Furthermore, the PDA/SF composite scaffolds could improve the structure and performance of SF for bone tissue regeneration.

Zhou et al. developed a multifunctional hydrogel scaffold through incorporating PDA-functionalized BG nanocomposites into F127-*ε*-Poly-L-lysine hydrogel (BG@PDA/FCB) for skin tumor treatment and wound healing [134]. The BG@PDA/FCB composite scaffolds were fabricated by a freeze-drying method and showed porous structures, which could effectively speed up wound healing *in vivo* by stimulating collagen deposition and angiogenesis. The BG@PDA/FCB composite scaffolds also exhibited high antitumor capability, suppressing melanoma growth *in vivo* via an efficient photothermal effect. After that, Zhou et al. reported polypyrrole@polydopamine (PPy@PDA)-functionalized poly(glycerol-amino acid)-based composite scaffolds (PGFP) [136]. The PGFP composite scaffolds loaded with DOX drugs exhibited significant photothermal chemotherapy effects *in vivo* and *in vitro* and at the same time accelerated the formation of granulation tissue, the deposition of collagen and the differentiation of vascular endothelium, promoting skin regeneration.

In addition to PDA and PPy, oligomeric proanthocyanidins (OPC), a kind of grape seed extract, can also be used as a natural PTA for tumor treatment and tissue regeneration by hybridization with 3D scaffolds. Ma et al. fabricated composite scaffolds of calcium silicate nanowires, sodium alginate and OPC through a 3D printing strategy for melanoma therapy and wound healing [137]. The composite scaffolds showed an ablation effect on melanoma cells and simultaneously promoted wound healing.

### 3.2. Scaffolds incorporating carbon-based nanomaterials

Carbon-based nanomaterials, such as carbon dots (CDs), GO, and SWCNTs, have attacted extensive attention as PTAs. They have been incorporated in 3D porous scaffolds of biodegradable polymers and bioceramics to combine their PTT effect and the tissue regeneration guiding effect of 3D scaffolds. Their composite scaffolds with chitosan/nanohydroxyapatite, polycaprolactone and β-tricalcium phosphate have been explored for possible applications to treat bone or skin cancer [138,139]. Lu et al. reported zero-dimensional CD-doped chitosan/nanohydroxyapatite scaffolds [140]. Under NIR laser irradiation, the CD-CS/nanohydroxyapatite scaffolds effectively prevented the proliferation of osteosarcoma UMR-106 cells *in vitro* and significantly suppressed bone tumor growth *in vivo*. The composite scaffolds also showed outstanding antibacterial ability against *S. aureus* and *E. coli*. Moreover, compared to the CS/nanohydroxyapatite scaffolds, the CD-CS/nanohydroxyapatite composite scaffolds enhanced the adhesion and osteoinductivity of MSCs *in vitro* through upregulating the related genes and effectively accelerated the formation of new bone tissue *in vivo*.

**Figure 5.**
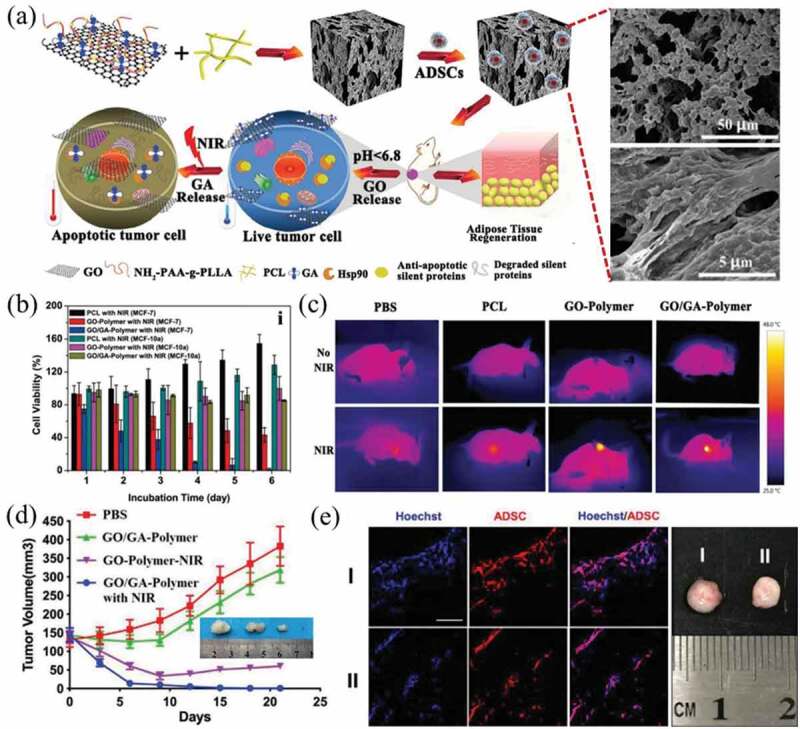
(a) Schematic illustration of the synthesis and therapeutic process of ADSC-loaded GO-GA-polymer scaffold for tumor selective low-temperature PTT and adipose tissue regeneration. (b) Cell viability of MCF-7 cells and MCF-10a cells incubated with PCL, GO-PCL, and GO-GA-PCL scaffolds for different times. (c) *In vivo* photothermal images of tumor-bearing mice. (d) Tumor growth curves after the implantation of different formulations. (e) Confocal fluorescence microscopy images of rat ADSCs and photographs of regenerated adipose tissue 2 months after implantation I) with and II) without NIR irradiation. Reproduced with permission [141]

Bai et al. developed a pH-responsive scaffold (GO-PCL) by hybridizing polycaprolactone (PCL), polyacrylic acid-g-polylactic acid (PAA-g-PLLA) and GO functionalized with gambogic acid (GA) (Figure 5(a)) [141]. In the slightly acidic environment surrounding cancer cells, GA could be released from the GO-PCL composite scaffolds and bind to Hsp90 protein, enabling mild-temperature PTT for cancer cells without affecting normal cells. The mild temperature PTT of the scaffolds could cause more than 95% cell death of breast cancer MCF-7 cells *in vitro* (Figure 5(b)), which further inhibited cancer growth and eventually eliminated the cancer cells *in vivo* (Figure 5(c, d)). Meanwhile, the GO-PCL composite scaffolds seeded with adipose-derived stem cells (ADSCs) significantly accelerated the formation of new adipose tissue (Figure 5(e)). Ma et al. fabricated a bifunctional GO-functionalized *β*-tricalcium phosphate composite scaffold (GO/TCP) by 3D printing combined surface modification strategies [26]. The as-prepared GO/TCP composite scaffolds could effectively ablate osteosarcoma MG63 cells *in vivo* and promote bone tissue repair in the bone defects of rabbits. Mauro et al. reported composite scaffolds of polycaprolactone microfiber (PCLmf) and GO nanosheets that were prepared by an electrospinning technique [142]. The PCLmf-GO composite scaffolds enhanced the adhesion and proliferation of cells and even allowed cancer-associated fibroblasts (CAF) capture. Moreover, the PCLmf-GO composite scaffolds displayed remarkable performance to capture cancer cells and CAF, and simultaneously implement photothermal ablation of the captured cancer cells in situ due to the PTT property of GO with its high NIR absorbance.

### 3.3. Scaffolds incorporating gold nanomaterials

**Figure 6.**
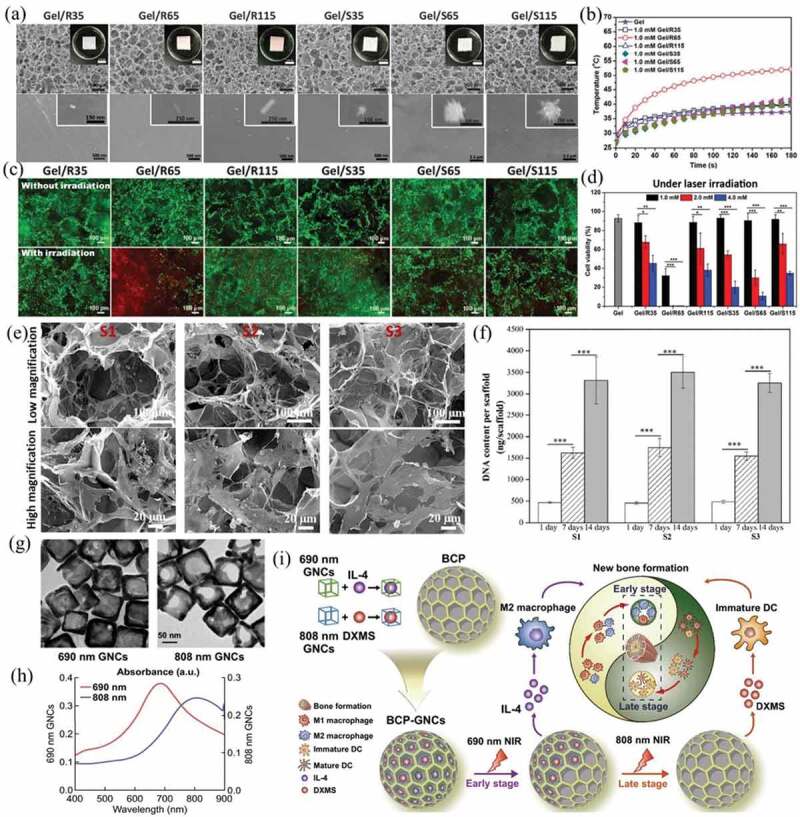
(a) Scanning electron microscopy (SEM) images and surface morphologies of Gel/R35, Gel/R65, Gel/R115, Gel/S35, Gel/S65 and Gel/S115 composite scaffolds prepared with 1.0 mM Au concentration. (b) Relation between temperature and irradiation time of different composite scaffolds. (c) Live/dead staining and (d) viability of HeLa cells in different composite scaffolds without or with laser irradiation. Reproduced with permission [143]. (e) SEM images of hMSC/scaffold constructs at low magnification and high magnification after hMSCs were cultured in the gelatin scaffold (S1), 2.0 mM (Au concentration) AuNR-gelatin scaffold (S2) and 4.0 mM (Au concentration) AuNR-gelatin scaffold (S3). (f) Quantification of the DNA content of hMSC/scaffold constructs after the hMSCs were cultured in S1, S2 and S3 for 1, 7 and 14 days. Reproduced with permission [146]. (g) TEM images of 690 nm and 808 nm gold nanocages (AuNCs). (h) Absorbance spectra of 690 nm AuNCs and 808 nm AuNCs. (i) Modification of BCP with drug-loaded AuNCs according to the immune response for promoting new bone formation. GNCs shown in the figure represent AuNCs. Reproduced with permission [147]

Due to the easy synthesis and good photothermal conversion capacity of gold nanoparticles, gold nanoparticles of different morphologies have been hybridized with gelatin and calcium phosphate to construct multifunctional composite scaffolds for combined effect of cancer cell killing effect and tissue regeneration promotion effect. Zhang et al. prepared composite scaffolds of gelatin and gold nanoparticles of different shapes and sizes (Figure 6(a)), including AuNRs and AuNSs of 35, 65, and 115 nm, to retain the gold nanoparticles in the tumor site at a high concentration and long period to achieve repeatable local heating [143]. The composite scaffolds showed different photothermal effects as the LSPR of gold nanoparticles changed with their shape and size. Among the composite scaffolds, those prepared with 65 nm AuNRs showed the highest photothermal conversion capability, increasing the temperature up to 50°C at 1 mM Au concentration (Figure 6(b)). The therapeutic effect of the composite scaffolds was confirmed by HeLa cells seeded on scaffolds and irradiated by a 805 nm laser (Figure 6(c,d)). Furthermore, the AuNR- or AuNS-based composite scaffolds were bound with folic acid, which was able to target folic acid receptor-positive tumor cells, to specifically capture tumor cells and improve the potential therapeutic outcome of the composite scaffolds [144]. In addition, dendritic cells could be activated by coculturing with ablated tumor cells in the composite scaffold, and multiple cytokines were secreted to induce tumor-specific immunotherapy [145].

In addition to the PTT effect to ablate cancer cells, the AuNRs/gelatin composite scaffolds promoted the proliferation and adipogenesis differentiation of human bone marrow-derived mesenchymal stem cells (hMSCs) [146]. Due to their high porosity and interconnectivity, the composite scaffolds could support cell adhesion and proliferation (Figure 6(e)), which was confirmed by culturing hMSCs in the scaffold for 14 days followed by DNA content quantification (Figure 6(f)). Importantly, the adipogenic differentiation of hMSc in the composite scaffolds was analyzed, and the positive results indicated that the AuNR-based composite scaffolds could exhibit multiple functions for both PTT and adipose regeneration.

Composite scaffolds of biphasic calcium phosphate (BCP) and gold nanocages have been prepared for controlled drug release. Zhao et al. prepared composite scaffolds of BCP and two types of gold nanocages that had LSPR peaks at 690 nm and 808 nm (Figure 6(g, h)) [147]. The gold nanocages were conjugated with thermally responsive 1-tetradecanol and loaded with different drugs (interleukin-4 or dexamethasone). The drug-loaded gold nanocages were incorporated into the BCP to form composite scaffolds. When the composite scaffolds were irradiated with 690 nm or 808 nm laser, they could release interleukin-4 and dexamethasone at early and late stages of osteoinduction, respectively, to adjust the local immune microenvironment for new bone formation (Figure 6(i)).

### 3.4. Scaffolds incorporating copper-based nanomaterials

**Figure 7.**
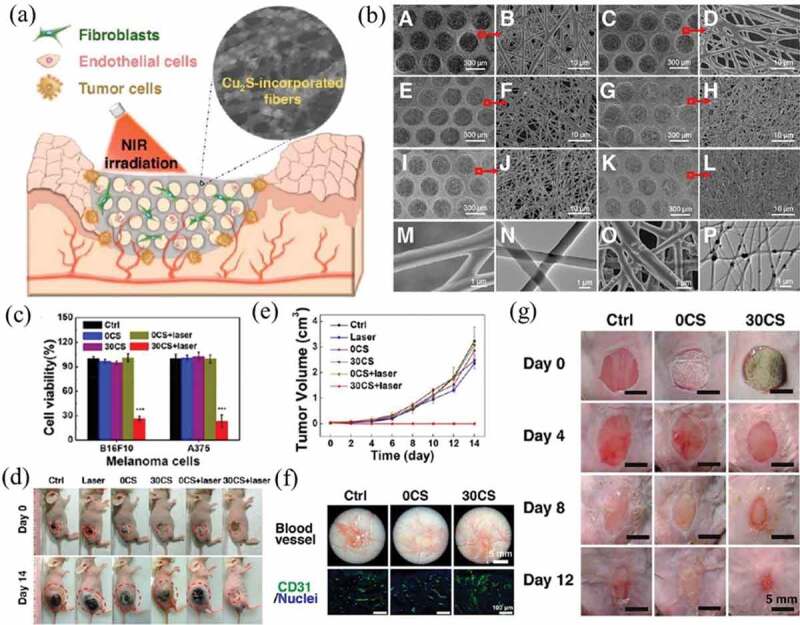
(a) Schematic illustration of the therapeutic process of Cu_2_S (CS)-PLA/PCL scaffold for cancer PTT and wound healing. (b) Representative SEM images of 0 wt% CS-PLA/PCL (A, B), 10 wt% CS-PLA/PCL (C, D), 20 wt% CS-PLA/PCL (E, F), 30 wt% CS-PLA/PCL (G, H), 40 wt% CS-PLA/PCL (I, J), and (K, L) 50 wt% CS-PLA/PCL membranes at different magnifications. Representative high-resolution SEM and TEM images of (M, N) 0 wt% CS- and (O, P) 30 wt% CS-PLA/PCL. (c) Cell viability of melanoma cells after different types of treatment. (d) Representative photographs of mice at day 0 and day 14 after various treatments. (e) Growth curve of skin cancer in different groups of mice after treatment. (f) Representative photographs of the wound beds showing angiogenesis (up) and immunofluorescence staining (down; green: CD31, blue: nuclei) in the wound healing region 12 days after treatment. (g) Representative skin wound photographs on day 0, 4, 8, and 12. Reproduced with permission [149]

Copper-based chalcogenides, such as CuS, Cu_2-x_S and Cu_2-x_Se, are effective PTAs. In addition, many studies have proven that Cu^2+^ can accelerate tissue repair through promoting cell migration, angiogenesis and collagen deposition [148]. Therefore, their composite scaffolds with biodegradable polymers have been prepared and investigated for a combined effect of PTT and tissue regeneration. Wang et al. fabricated a dual-functional scaffold by incorporating Cu_2_S nanoflowers into poly(D, L-lactic acid)/poly (*ε*-caprolactone) polymer blend (PLA/PCL) fibers by an electrospinning method (Figure 7(a, b)) [149]. Cu_2_S nanoflowers served not only as a local PTA for cancer treatment but also as a Cu^2+^ source for wound healing. Under NIR laser irradiation, the Cu_2_S-PLA/PCL composite scaffolds exhibited controllable photothermal performance, caused a high death rate of skin tumor B16F10 cells *in vitro* (Figure 7(c)), and effectively inhibited tumor growth *in vivo* (Figure 7(d, e)). The composite scaffolds also promoted the adhesion, proliferation and migration of normal skin cells and significantly accelerated angiogenesis and the healing of full-thickness skin defects *in vivo* (Figure 7(f, g)). Dong et al. reported BG scaffolds modified with CuFeSe_2_ nanocrystals that could significantly inhibit bone tumor growth *in vivo*, ultimately promoting the formation of new bone [150].

In addition to copper sulfide and its copper-deficient structures, molybdenum disulfide (MoS_2_) is another typical metal sulfide that has been incorporated into 3D scaffolds for PTT and tissue engineering applications. Wang et al. constructed an integrated scaffold by fixing a PLGA-functionalized MoS_2_ film on the surface of BG [151]. The MoS_2_-integrated BG composite scaffolds could rapidly elevate temperature to ablate osteosarcoma cells and inhibit tumor growth *in vivo* under 808 nm laser irradiation. Meanwhile, the composite scaffolds could stimulate the proliferation and differentiation of rat MSCs, upregulate the expression of osteogenesis-related genes and promote bone repair. Wang et al. also reported 3D-printed bioceramic scaffolds with MoS_2_ nanosheets for inhibiting cancer growth and inducing bone tissue repair *in vivo* [152].

Silicate-based biomaterials have been reported to promote epithelial regeneration and collagen deposition during wound healing [153]. Accordingly, Yu et al. prepared electrospun scaffolds doped with copper silicate hollow microspheres (CSHMSs), where the CSHMSs were used as drug carriers, as a PTA for the treatment of melanoma, and as a source of therapeutic elements (copper and silicon) for local wound healing [154]. CSHMSs were first synthesized by a hydrothermal method and then electrospun into the PLA/PCL matrix to obtain CS-PLA/PCL composite scaffolds. The CS-PLA/PCL scaffolds exhibited synergistic effects of PTT and chemotherapy to kill skin cancer cells both *in vitro* and *in vivo*, promoted the adhesion and proliferation of normal skin cells, and accelerated skin wound healing. By a similar approach, Yu et al. integrated CaCuSi_4_O_10_ NPs into electrospun PLA/PCL scaffolds for tumor treatment and skin tissue regeneration [155]. The composite scaffolds quickly ablated tumor cells under NIR laser irradiation, effectively inhibited tumor growth in mice, and promoted wound healing by releasing bioactive Cu^2+^ and SiO_4_^4-^ ions from the CaCuSi_4_O_10_ NPs after cancer treatment.

### 3.5. Scaffolds incorporating iron-based nanomaterials

**Figure 8.**
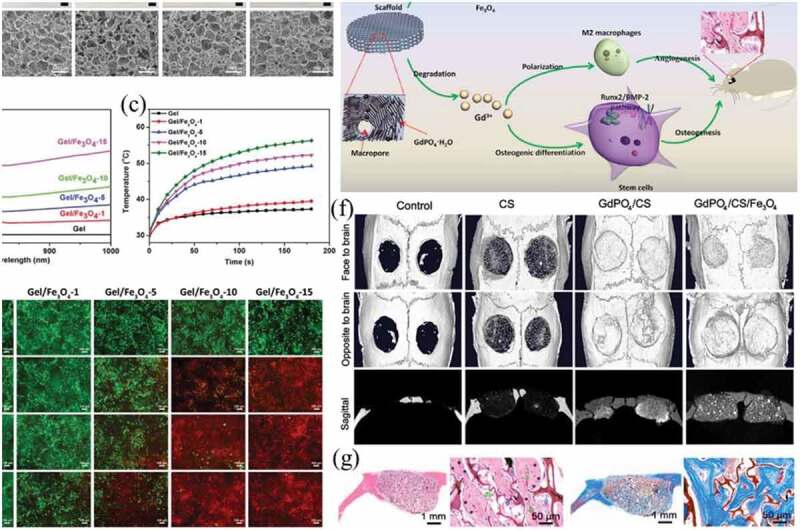
(a) Gross appearance and SEM images of Gel scaffold and Gel/Fe_3_O_4_ composite scaffolds prepared with different concentration of Fe_3_O_4_ nanoparticles. (b) UV-Vis absorption spectra of Gel scaffold and Gel/Fe_3_O_4_ composite scaffolds. (c) Relation between temperature and irradiation time of Gel scaffold and Gel/Fe_3_O_4_ composite scaffolds under NIR laser irradiation. (d) Live/dead staining of HeLa cells in Gel scaffold and Gel/Fe_3_O_4_ composite scaffolds without or with NIR laser irradiation for different cycles. Reproduced with permission [157]. (e) Schematic illustration of GdPO_4_/CS/Fe_3_O_4_ scaffolds for NIR-induced photonic hyperthermia and enhanced bone tissue regeneration. (f) Micro-CT images of calvarial defect repair model at 3 months after surgery for the control, CS, GdPO_4_/CS, GdPO_4_/CS/Fe_3_O_4_ groups. (g) Newly formed bones were detected in the GdPO_4_/CS/Fe_3_O_4_ by H&E staining and Masson’s trichrome staining. Reproduced with permission [158]

Iron nanomaterials can exhibit plasma performance and generate hyperthermic effects in the presence of NIR light and alternating magnetic fields. Previous studies have also proven that Fe^3+^ ions can affect the differentiation and gene expression of MSCs [156]. Therefore, magnetic iron oxide nanoparticles have been incorporated into 3D scaffolds to investigate their PTT and magnetothermal heating (MTH) effects. Zhang et al. recently fabricated 3D composite scaffolds of Fe_3_O_4_ and gelatin (Gel/Fe_3_O_4_) for the local photothermal ablation of tumor cells (Figure 8(a)) [157]. The Gel/Fe_3_O_4_ composite scaffolds had significant absorption in the NIR region, and under NIR laser irradiation, the local temperature increased rapidly (Figure 8(b, c)). The Gel/Fe_3_O_4_ composite scaffolds exhibited the repeated local photothermal ablation of HeLa cells and suppressed the recurrence rate of tumor cells (Figure 8(d)). For bone metastases caused by breast cancer, after surgical removal, the remaining cancer cells will usually induce cancer recurrence. Moreover, surgical removal can cause local bone defects. Zhao et al. fabricated multifunctional Fe_3_O_4_/GdPO_4_/CS scaffolds in which Fe_3_O_4_ NPs and GdPO_4_ nanorods were incorporated into a chitosan (CS) matrix (Figure 8(e)) [158]. The Fe_3_O_4_ NPs in the Fe_3_O_4_/GdPO_4_/CS composite scaffolds enhanced NIR absorption capacity, and the local temperature rose high enough to induce cancer cell apoptosis under 808 nm laser irradiation, which effectively avoided cancer recurrence. Moreover, the GdPO_4_ nanorods in the Fe_3_O_4_/GdPO_4_/CS composite scaffolds stimulated the BMP-2/Smad/RUNX2 signaling pathway, thereby promoting cell proliferation and bone reconstruction (Figure 8(f, g)). Similarly, magnetic SrFe_12_O_19_ NP-modified bioactive chitosan porous scaffolds were fabricated for bone reconstruction and PTT against cancers [159].

In addition, the MTH effect of the composite scaffolds has been confirmed for PTT of cancers. Zhang et al. reported 3D-printed *β*-tricalcium phosphate bioceramic scaffolds with surface-functionalized Fe_3_O_4_/GO composite layers (TCP/Fe_3_O_4_/GO) [160]. The TCP/Fe_3_O_4_/GO composite scaffolds had a relatively consistent hole structure with a diameter of approximately 300–500 µm, and the temperature could be adjusted by controlling the intensity of the magnetic field and the content of Fe_3_O_4_ NPs. The TCP/Fe_3_O_4_/GO scaffolds with an excellent MTH capacity induced more than 75% osteosarcoma cell death and significantly stimulated alkaline phosphatase (ALP) activity and osteogenic gene expression. Recently, Dong et al. constructed a multifunctional akermanite scaffold (AKT/Fe_3_O_4_/CaO_2_) by loading CaO_2_ and Fe_3_O_4_ nanoparticles [161]. The loaded CaO_2_ nanoparticles acted as a H_2_O_2_ source to achieve self-sufficient H_2_O_2_ nanocatalytic osteosarcoma treatment though a Fenton-like Fe_3_O_4_ reaction and provided Ca^2+^ ions to enhance bone regeneration. The AKT/Fe_3_O_4_/CaO_2_ composite scaffolds with synergistic magnetothermal and nanocatalytic therapy effects significantly suppressed tumor growth and enhanced the bone regeneration by releasing Ca^2+^.

### 3.6. Scaffolds incorporating black phosphorus

**Figure 9.**
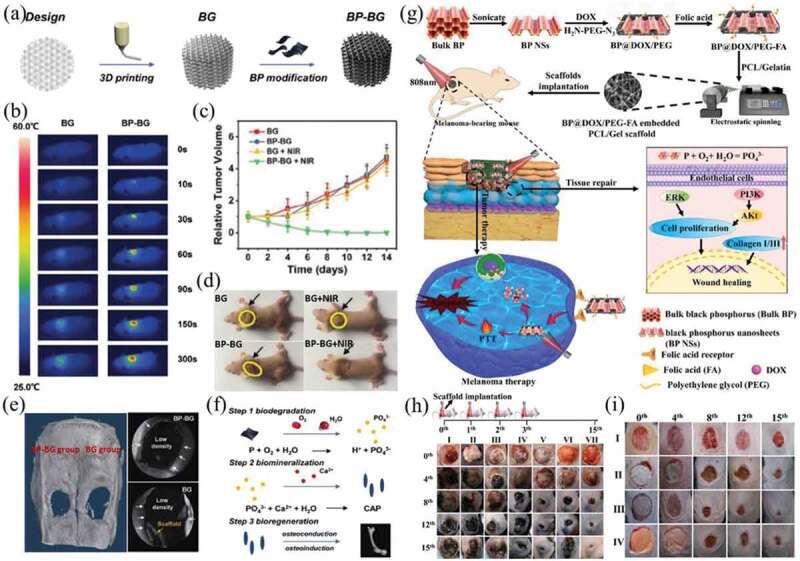
(a) Preparation scheme of BP-BG composite scaffolds. (b) Infrared thermographic photographs of osteosarcoma-bearing nude mice implanted with BG and BP-BG scaffolds. (c) Tumor growth curves of mice after different treatments. (d) Photographs of osteosarcoma-bearing mice after different treatments on day 14. (e) Micro-CT imaging after treatment for 8 weeks. The defect areas were implanted with BP-BG and BG scaffold, respectively. (f) Stepwise therapeutic strategy for the elimination of osteosarcoma followed by osteogenesis in BP-BG composite scaffolds. Reproduced with permission [162]. (g) Preparation scheme of nanofibrous scaffolds and the underlying mechanisms of PTT/chemotherapy and tissue regeneration. (h) Representative images of the tumors on days 0, 4, 8, 12 and 15 after treatment with control (I), GP (II), GP+laser (III), 5BP-PEG-FA-GP (IV), 5BP-PEG-FA-GP+laser (V), 5BP@DOX-PEG-FA-GP (VI) and 5BP@DOX-PEG-FA-GP+laser (VII). (i) Representative images of dermal wounds in different treatment groups on days 0, 4, 8, 12 and 15. The treatments include control (I), GP(II), 5BP-PEG-FA-GP (III) and 5BP@DOX-PEG-FA-GP (IV). Reproduced with permission [163]

Phosphorus is one of the important elements that make up human bone, accounting for approximately 1% of the total weight of the human body. Because of their interactions with oxygen, visible light and water, BP nanomaterials are easily oxidized and degraded in aqueous solution to form phosphates and phosphonates, thereby causing bone formation and osseointegration. The biodegradation, good biocompatibility and excellent PCE of BP nanomaterials make them attractive components of composite scaffolds for combined effect of PTT and tissue regeneration. Yang et al. incorporated BP nanosheets into 3D-printed BG scaffolds (BP-BG, Figure 9(a)) for localized osteosarcoma therapy and bone tissue regeneration [162]. Due to the excellent tissue regeneration capability and remarkable PTT effects, the BP-BG composite scaffolds exhibited good photothermal ablation against osteosarcoma-bearing nude mice at the early stage after implantation (Figure 9(b-d)) and stimulated osteogenesis, osteoinduction and osteoconduction at the late stage of implantation (Figure 9(e)). Finally, the BP NSs in the scaffolds were oxidized and released PO_4_^3-^ to form calcium phosphate (CAP), which induced biomineralization for bone regeneration (Figure 9(f)).

The potential effect of BP NSs for enhancing skin regeneration has been explored [163]. The composite scaffolds were prepared by embedding BP-DOX-FA into gelatin-polycaprolactone (PCL) nanofibrous scaffolds for melanoma and skin regeneration (Figure 9(g)). *In vivo* and *in vitro* studies showed that the composite scaffolds could selectively kill melanoma cells through cooperative PTT and heat-triggerable DOX chemotherapy (Figure 9(h)), and the BP NSs promoted skin tissue regeneration by activating the PI3K/Akt and ERK1/2 pathways (Figure 9(i)).

Recently, Qian et al. explored the effect of BP NSs on the restoration of neurogenesis, angiogenesis and immune homeostasis. The composite scaffolds were produced by using integrative layer-by-layer assembly [164]. The BP NSs were prepared in a PCL dichloromethane solution and sprayed from multiple nozzles onto the conduit-shaped mold. To produce pore alignment, the flip microneedle arc panel was buttoned onto the BP/PCL layer, and staggered pores were added to the scaffolds. The spraying process was repeated to prepare the outermost layer. The pore structure of the scaffolds facilitated the exchange of oxygen and water for axon sprouting. The composite scaffolds allowed controlled BP release to achieve a low-ROS microenvironment and enhance angiogenesis for nerve regeneration. BP at concentrations up to 0.5% was electrically conductive and could regenerate peripheral nerve defects.

### 3.7. Scaffolds incorporating MXenes

**Figure 10.**
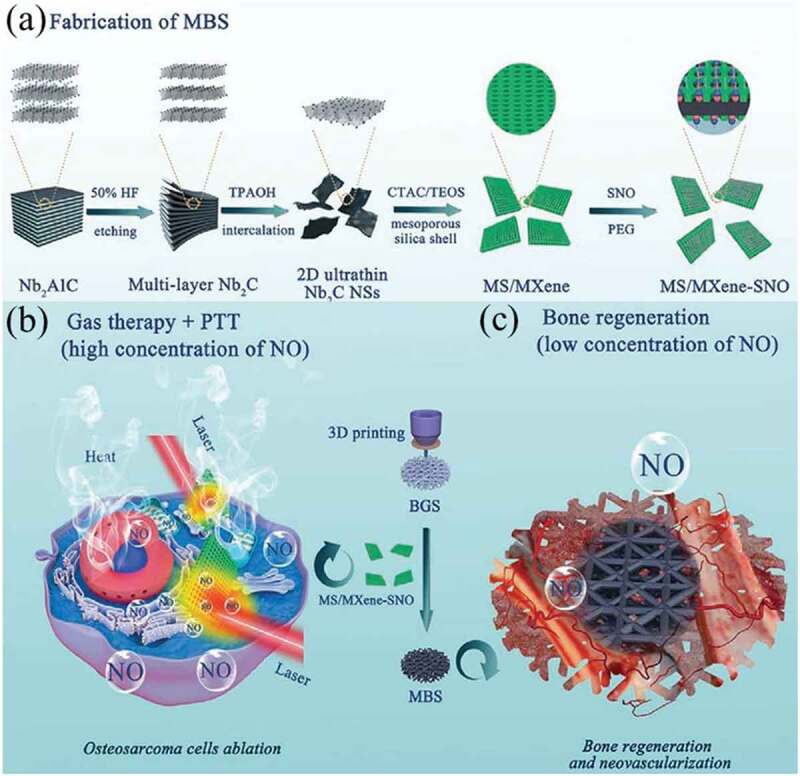
(a) Preparation scheme of multifunctional mesoporous silica/MXene-SNO (MBS). (b) Combination of PTT and gas therapy for cancer therapy. (c) Promotion of bone regeneration in composite scaffolds with a low concentration of NO (c). Reproduced with permission [167]

MXenes such as Nb_2_C possesses good biocompatibility and excellent PCE, which meets the requirement for scaffolds used for tumor treatment and tissue repair. In addition, nitric oxide (NO) has been extensively applied in medicine for its pro-angiogenic, antibacterial, and anticancer effects [165,166]. They have been hybridized with BG and titanium to construct composite scaffolds for cancer therapy and tissue regeneration. Yang et al. fabricated a multifunctional BG scaffold by the integration of Nb_2_C wrapped with S-nitrosothiol (NO donor)-grafted mesoporous silica with 3D printing BG scaffolds (Nb_2_C/MS/BG) (Figure 10(a-c)) [167]. The Nb_2_C/MS/BG composite scaffolds could produce NIR-II triggered photonic hyperthermia from Nb_2_C and perform precisely controlled NO release for the cooperative multitarget treatment of bone cancer. The phosphorus and calcium components from the degradation of the Nb_2_C/MS/BG composite scaffolds significantly promoted the biological activity of bone regeneration, which was further enhanced by NO through triggering adequate blood supply. Similarly, a Nb_2_C titanium plate (Nb_2_C@TP) composite scaffold was developed to eliminate bacterial infection and promote tissue regeneration [168]. The Nb_2_C@TP-based hyperthermia therapy not only effectively ablated bacteria *in vivo* but also mitigated excessive inflammatory responses and ROS production, thereby stimulating angiogenesis and tissue repair.

Pan et al. reported the integration of 2D Ti_3_C_2_ MXenes with a 3D-printed BG scaffold (Ti_3_C_2_-BG) for bone cancer treatment and reconstruction [169]. The Ti_3_C_2_-BG composite scaffolds not only killed the bone cancer cells based on their photothermal properties but also induced the differentiation of human bone marrow MSCs into osteoblasts, which accelerated bone repair *in vivo*. In the process of bone tissue reconstruction, the Ti_3_C_2_-BG composite scaffolds gradually degraded to provide necessary minerals (such as titanium-based species) and space for the newly formed bone tissue.

### 3.8. Scaffolds incorporating other nanomaterials

MOFs, such as copper-coordinated tetrakis(4-carboxyphenyl) porphyrin (Cu-TCPP), have also been embedded in 3D scaffolds to produce an excellent photothermal response to NIR laser irradiation while maintain the tissue regeneration capacity of the 3D scaffolds. Dang et al. reported a Cu-TCPP nanosheet interface-structured *β*-tricalcium phosphate (TCP) scaffold (Cu-TCPP/TCP) for cancer therapy and bone reconstruction [170]. The Cu-TCPP/TCP composite scaffolds ablated bone cancer cells and suppressed their growth by exerting photothermal effects in a mouse model. The composite scaffolds also promoted the osteogenic and angiogenic differentiation of hBMSCs and HUVECs. After implantation into the bone defects of rabbits, they effectively boosted bone tissue repair.

Bi has a small carrier effective mass and long Fermi wavelength and has there attracted attention for hybridization with porous scaffolds for biomedical applications. Wang et al. fabricated a novel model of Bi_2_O_3_-doped BG with bioactivity and photothermal response for bone cancer therapy and tissue regeneration [171]. Both *in vitro* and *in vivo* studies demonstrated that Bi_2_O_3_-doped BG efficiently destroyed bone cancer cells under 808 nm laser irradiation, and simultaneously facilitated the multiplication, differentiation and mineralization of osteoblasts on their surface, which promoted the regrowth of the bone tissue.

Various bioceramics, such as calcium phosphate-based bioceramics, silicate bioceramics and BGs, have been proven to have good activity for bone tissue regeneration and tooth restoration [172,173]. Wang et al. synthesized black bioceramics through the magnesium thermal reduction of traditional white ceramics, including silicate-based (CaSiO_3_, MgSiO_3_) and phosphate-based (Ca_3_(PO_4_)_2_, Ca_5_(PO_4_)_3_(OH)) ceramics [174]. *In vitro* and *in vivo*, these black bioceramics not only showed excellent antitumor effects against both melanoma and osteosarcoma but also significantly promoted skin/bone tissue repair.

## 4. Conclusions and future perspectives

In this review, we systematically summarized the commonly used PTAs in PTT applications and their composite scaffolds for cancer therapy and tissue regeneration applications (Tables 1 and 2). Unlike traditional cancer treatments, these composite scaffolds can be effectively implanted into tumor sites for the photothermal ablation of tumor cells, and their pore structures benefit cell growth and tissue repair after tumor treatment. Meanwhile, the elements released from the composite scaffolds (such as calcium, copper, and phosphorus) have stimulating and inducing effects on the adhesion, proliferation, and differentiation of cells, which can promote tissue regeneration. In addition, synergistic therapy strategies have been developed in composite scaffolds for improving treatment efficacy or reducing side effects.

Despite the successful development of multifunctional composite scaffolds for tumor therapy and tissue regeneration applications, there are still some challenges in clinical applications. First, the tissue penetration depth of NIR light is a limitation of PTT. The photothermal effect of PTAs can effectively inhibit the growth of superficial tumors such as melanoma, but for deep tumors such as gastrointestinal stromal tumors, its inhibitory effect may be limited. To solve this problem, the exploration of new NIR-II biowindow or magnetothermal agents is necessary. Second, the biodegradation behavior of composite scaffolds *in vivo* and their potential long-term toxicity should be clarified. Although some biomaterials used in the construction of scaffolds can enhance the physiological stability or biocompatibility, the biodistribution and metabolic pathways of the composite scaffolds still need systematic investigation. Third, synergistic therapy strategies with composite scaffolds should be further developed. To date, synergistic therapy via using composite scaffolds, such as synergistic PTT/chemotherapy, PTT/PDT and PTT/gas therapy, has successfully enhanced tumor therapeutic efficacy, but there are still other therapeutic modalities that can be collaboratively developed and utilized, such as immunotherapy, sonodynamic therapy and starvation therapy. Fourth, since actual tumor and regeneration animal models are difficult to obtain, it is challenging to evaluate the processes and mechanisms of tumor treatment and tissue regeneration *in vivo*. For example, the tumor inhibition and bone tissue reconstruction effects of composite scaffolds used to treat bone tumors are usually studied separately, which cannot imitate the actual situation in disease treatment. Therefore, choosing a suitable animal model is of great importance to advance the clinical application of these composite scaffolds.

Currently, a variety of nanomaterials with engineering scaffolds have been developed for cancer therapy and tissue regeneration. Nevertheless, most of these composite scaffolds are in the preclinical stage, and further research still needs to be done to promote their clinical applications.
